# Exosomes Derived from Human Umbilical Cord Mesenchymal Stem Cells Relieve Acute Myocardial Ischemic Injury

**DOI:** 10.1155/2015/761643

**Published:** 2015-05-27

**Authors:** Yuanyuan Zhao, Xiaoxian Sun, Wenming Cao, Jie Ma, Li Sun, Hui Qian, Wei Zhu, Wenrong Xu

**Affiliations:** ^1^School of Medical Science and Laboratory Medicine, Jiangsu University, Zhenjiang, Jiangsu 212013, China; ^2^The Affiliated Hospital, Jiangsu University, Zhenjiang, Jiangsu 212001, China

## Abstract

This study is aimed at investigating whether human umbilical cord mesenchymal stem cell- (hucMSC-) derived exosomes (hucMSC-exosomes) have a protective effect on acute myocardial infarction (AMI). Exosomes were characterized under transmission electron microscopy and the particles of exosomes were further examined through nanoparticle tracking analysis. Exosomes (400 *μ*g protein) were intravenously administrated immediately following ligation of the left anterior descending (LAD) coronary artery in rats. Cardiac function was evaluated by echocardiography and apoptotic cells were counted using TUNEL staining. The cardiac fibrosis was assessed using Masson's trichrome staining. The Ki67 positive cells in ischemic myocardium were determined using immunohistochemistry. The effect of hucMSC-exosomes on blood vessel formation was evaluated through tube formation and migration of human umbilical vein endothelial cells (EA.hy926 cells). The results indicated that ligation of the LAD coronary artery reduced cardiac function and induced cardiomyocyte apoptosis. Administration of hucMSC-exosomes significantly improved cardiac systolic function and reduced cardiac fibrosis. Moreover, hucMSC-exosomes protected myocardial cells from apoptosis and promoted the tube formation and migration of EA.hy926 cells. It is concluded that hucMSC-exosomes improved cardiac systolic function by protecting myocardial cells from apoptosis and promoting angiogenesis. These effects of hucMSC-exosomes might be associated with regulating the expression of Bcl-2 family.

## 1. Introduction

Myocardial infarction (MI) usually results in irreversible myocardial cell loss and heart function failure due to the restricted blood supply and is one leading cause of death worldwide. Although many treatments have traditionally been used, damaged myocardial cells remain nonrenewable and scarred myocardial tissue still cannot be restored. It has been indicated that stem cell therapy in the acute phase of MI prevents cardiomyocytes apoptosis, promotes local neoangiogenesis, improves myocardial perfusion, and reduces the local inflammatory response [1–3]. In the late phase of MI, cell therapy can replace the dead myocardium with viable cardiomyocytes, smooth muscle cells, and endothelial cells, to reduce scar formation [4, 5]. Therefore, transplantation of stem cells following AMI has been tested in clinical trials [6, 7].

Mesenchymal stem cells (MSCs) are considered as the lead candidates for cellular therapy. MSCs are multipotent and have been demonstrated to improve cardiac function following intramyocardial transplantation into infarcted myocardium [8–11]. MSCs are broadly distributed throughout the body [12] outside bone marrow and reside in adipose tissue, gut, lung, liver, placenta, amniotic fluid, dental pulp, and periodontal ligament [13, 14]. The cells most commonly used in clinical trials to date originate from bone marrow, adipose tissue, and umbilical cord. hucMSC is more suitable for the treatment of AMI because fewer ethical issues are involved; they have high self-renewal ability and low immunogenicity [15, 16]. Our research team has previously reported the potential curative effect of hucMSC and bone marrow MSC on repairing the liver and kidney [17–20].

Rota et al. [21] have reported how transplanted bone marrow cells regenerated myocardium from engraftment in the infarcted myocardium to form a functional myocyte. Donor cell-derived myocytes exhibited electrical characteristics similar to spared myocytes but showed a prolongation of the AP and enhanced cell shortening. It has also been indicated that MSCs became actin-positive and formed gap junctions with the native myocytes when MSCs were cocultured with ventricular myocytes [22]. The results of several* in vivo* studies support the possibility that transplanted MSCs could contribute to regeneration through direct engraftment and differentiation. However, cardiomyogenic differentiation is often not observed or at best occurs at extremely low levels. Based on two studies, only 0.5–5% of the engrafted cells have been reported to differentiate [23, 24].

Gnecchi et al. found that soluble paracrine factors can promote myocardial regeneration and angiogenesis [25]. The soluble paracrine factors can also recruit bone marrow cells and cardiac stem cells to the myocardial injury area [26]. However, it is not easy to identify which paracrine factor(s) play critical roles in AMI treatment because of the diversity and complexity of the paracrine factors [25]. Using conditioned medium from human embryonic MSC, Timmers et al. found that only factors which are greater than 1,000 kDa had the ability to repair myocardial ischemia-reperfusion injury in a mouse model. Their further research verified that these factors are exosomes released from MSCs [27]. MSC-derived exosomes were also investigated in a mouse model of ischemia/reperfusion injury [28]. Exosomes are the most effective active paracrine ingredients, playing an important role in cell to cell communication, which have great potential in repair of the damaged tissue [28, 29].

Our studies have also shown that hucMSC-exosomes eased liver fibrosis induced by CCl_4_ [30], protected against cisplatin-induced renal oxidative stress and apoptosis [31], and enhanced cutaneous wound healing [32]. However, whether hucMSC-exosomes can ease myocardial injury and improve cardiac function remains unknown. In this study, hucMSC-exosomes were injected into Sprague-Dawley (SD) rats immediately via the tail vein after induction of AMI. Our study indicates that hucMSC-exosomes may promote ischemia myocardium regeneration.

## 2. Materials and Methods

### 2.1. Cell Culture

hucMSCs were isolated and cultured following the established method [33]. All people provided informed consent for the use of the cord in this experimental study, which was approved by the ethical committee of School of Medical Science and Laboratory Medicine, Jiangsu University, China. The hucMSCs were cultured in low glucose Dulbecco's modified Eagle's medium (L-DMEM) containing 10% fetal bovine serum (FBS) (Gibco, Grand Island, USA) at 37°C in humidified air with 5% CO_2_. The rat myocardial cells H9C2(2-1) and human umbilical vein endothelial cells (EA.hy926) were purchased from Shanghai cell bank, Chinese Academy of Medical Sciences. They were cultured in high glucose Dulbecco's modified Eagle's medium (H-DMEM) containing 10% FBS under 37°C in humidified air with 5% CO_2_.

### 2.2. Extraction, Purification, and Characterization of hucMSC-Exosomes

**Figure 1:**
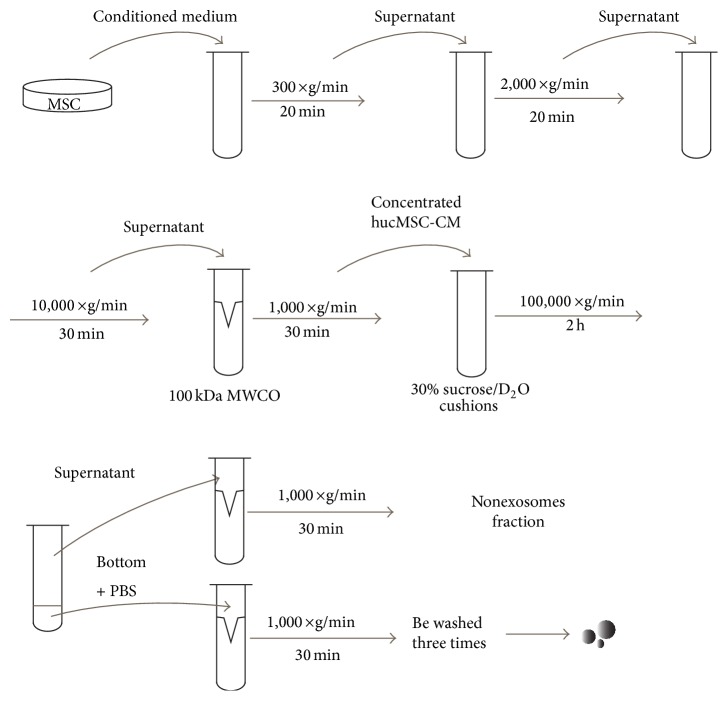
Separation process of exosomes derived from hucMSC.

The exosomes were isolated following the procedure described by Qu et al. [34] with minor modifications (Figure 1). In brief, the 10% FBS L-DMEM was replaced with 10% exosome-free FBS L-DMEM when cultured hucMSC reached 80–90% density. Exosome-free FBS was obtained by ultracentrifuge FBS at 100,000 ×g for 16 h. It was confirmed without exosomes in exosome-free FBS using NTA. The conditioned medium of hucMSC (hucMSC-CM) was collected after cells were cultured with exosome-free FBS L-DMEM for 48 hours. hucMSC-CM was centrifuged at 300 ×g for 20 min, 2,000 ×g for 20 min, and 10,000 ×g for 30 min to remove dead cells and cell debris. The hucMSC-CM was then concentrated using a 100 kDa molecular weight cut-off (MWCO) hollow fiber membrane (Millipore, USA) at 1,000 ×g for 30 min. The concentrated hucMSC-CM was loaded onto 5 mL 30% sucrose/D_2_O cushions and ultracentrifuged at 100,000 ×g for 2 hours (optimal-90k, Beckman Coulter, USA). The supernatant of the cushion was collected as nonexosome fraction and concentrated using 100 kDa MWCO centrifuge tube. The bottom of the cushion containing the exosomes was collected and washed three times with phosphate buffered saline (PBS) using 100 kDa MWCO centrifuge tube at 1,000 ×g for 30 min. The protein content of the nonexosome fraction and exosomes was determined using a BCA kit (CWBIO, Beijing, China). The nonexosome fraction and exosomes were filtered through 0.22 *μ*m membrane filter (Millipore, USA) and stored at −80°C for later use.

To characterize hucMSC-exosomes, a suspension of purified exosomes (20 *μ*L) was dropped onto a Formvar/carbon-coated grid and negatively dyed in 3% (w/v) phosphotungstic acid solution (pH 6.8) for 5 min. The morphology of the exosomes was observed under a transmission electron microscope (FEI Tecnai 12, Philips, Netherlands). Nanoparticle tracking analysis (NTA) was performed to analyze the particles of exosomes using a digital microscope LM10 system (NanoSight, Amesbury, UK). Video images of the movement of particles under Brownian motion were analyzed using NTA analytical software, and the particle concentration was also recorded.

### 2.3. Creation and Verification of AMI Model

Animal protocol was approved by Animal Experimental Center of Jiangsu University, Zhenjiang, China. Male SD rats, 220–250 g, were anesthetized using 10% chloral hydrate (Sinopharm Chemical Reagent Company, Shanghai, China) (300 mg/kg) by intraperitoneal injection and mechanically ventilated (Alcott Biotech Company, Shanghai, China). An AMI model was established by opening the thorax, extruding the heart and quickly and accurately ligating the left anterior descending (LAD) coronary artery with 6-0 suture. Finally the chest was closed by tightening the double purse suture. These rats were killed at 2 days, 1 week, and 4 weeks after surgery based on the different experiments. To confirm whether LAD coronary artery ligation could induce AMI, electrocardiogram was detected in the rats with LAD ligation as early as 24 h. TTC staining was performed at 48 h after surgery and echocardiograph was performed at 1 week after surgery. To investigate the therapeutic effect of hucMSC-exosomes, TUNEL staining and Ki67 immunohistochemistry were processed at 1 week after surgery. The echocardiography and Masson's trichrome staining were performed at four weeks after surgery.

To determine whether a rat AMI model was successfully created, electrocardiograms were recorded after ligation of the LAD coronary artery for 24 h using a biological signal recording system (10T, PowerLab, ADInstruments, Shanghai, China). Then, some of the rats were sacrificed using an overdose of anesthetic. Their thoraxes were opened and the hearts were removed and immersed in physiological saline. The hearts were cut into 5 transverse sections of the short axis of the left ventricles below the artery ligation site and soaped in 1% TTC dye (Sigma, USA) at 37°C for 15 min. The size of infarcted myocardium was measured. Echocardiography of other rats was performed to detect the cardiac function using a high frequency color ultrasound instrument (Vevo2100, VisualSonics, Canada) at 1 week after surgery.

### 2.4. Infusion of hucMSC-Exosomes and Evaluation of Cardiac Function

To investigate the effect of hucMSC-exosomes, rats were divided into three groups with six rats in each group. AMI + exosomes group: hucMSC-exosomes (400 *μ*g protein) suspended in 200 *μ*L PBS were infused via the tail vein. AMI + PBS group: 200 *μ*L PBS was infused via the tail vein. The sham group underwent neither LAD coronary artery ligation nor exosome infusion except that their chests were opened and the sutures were passed under the LAD coronary artery. In order to further confirm the effect of exosomes, hucMSC-CM without exosomes was also used in this study.

Echocardiography was performed 4 weeks after exosomes or nonexosome fraction infusion. The related parameters were recorded: left ventricular internal diameter at the end of diastole (LVID;d), left ventricular internal diameter at the end of systole (LVID;s), left ventricular volume at the end of diastole (LV Vol;d), left ventricular volume at the end of systole (LV Vol;s), left ventricular ejection fraction (LVEF), and left ventricular shortening fraction (LVFS).

### 2.5. TUNEL Assay, Infarction Size Measurement, and Immunohistochemistry

The number of apoptotic cells in the ischemic zone was measured by the TUNEL assay using an in situ cell apoptosis detection kit (Boster) according to the manufacturer's instructions. The microscopic ten areas were recorded and the TUNEL-positive cells were calculated by Image-Pro Plus 6.0.

For immunohistochemistry, the tissue sections were incubated with diluted primary antibodies against rat Ki67 (1 : 200) (Santa Crus) after blocking the nonspecific areas with 5% bovine serum albumin. Then, the tissue sections were incubated with biotin-conjugated anti-rabbit immunoglobulin G and streptavidin biotin and colored with 3,3′-diaminobenzidine. Finally the myocardial tissues in the border zone were photographed after staining with haematin. The cells positive for Ki67 were counted by Image-Pro Plus 6.0.

The myocardial tissue below the ligation position was fixed in 4% paraformaldehyde, embedded in paraffin and cut into 5 *μ*m serial sections which were then used for Masson's trichrome staining. Images were captured using a stereo microscope (SMZ-168, Motic, China) and were analyzed using Image-Pro Plus 6.0.

### 2.6. Hypoxic Experiments* In Vitro*

In order to study whether hucMSC-exosomes protected H9C2(2-1) cells against hypoxic injury, three experimental groups were designed as follows. Normoxia group: H9C2(2-1) cells were cultured in H-DMEM containing 10% FBS at 37°C in humidified air with 5% CO_2_. Hypoxia + exosomes group: H9C2(2-1) cells were cultured in H-DMEM containing 0.2% FBS and hucMSC-exosomes (200 *μ*g/mL) at 37°C in humidified air with 5% CO_2_, 2% O_2_, and 93% N_2_. Hypoxia + PBS group: H9C2(2-1) cells were cultured under the same conditions as the hypoxia + exosomes group except that the hucMSC-exosomes (200 *μ*g/mL) were replaced by isometric PBS. Twelve hours later, protein was extracted from the cells to detect expression of Bcl-2 family members.

### 2.7. Western Blot

hucMSC-exosomes or H9C2(2-1) cells were lysed in radioimmunoprecipitation assay (RIPA) buffer and phenylmethanesulfonyl fluoride (PMSF). The protein concentration was determined using the BCA protein assay kit. Equal quantities of protein were loaded and run on 12% SDS gels and then transferred onto PVDF membranes (Millipore, USA). The membranes were blocked with 5% skimmed milk for 1 h and incubated with diluted primary antibodies [CD9 (1 : 500; Bioworld), CD63 (1 : 300; SAB), Bcl-2 (1 : 500; Bioworld), Bax (1 : 500; Bioworld), and GAPDH (1 : 2,000; CWBIO)] at 4°C overnight. The membranes were incubated in goat anti-rabbit or anti-mouse antibodies (1 : 2,000; Bioworld) at 37°C for 1 h. The target proteins were detected using the Luminata Crescendo Western HRP substrate (Millipore, USA) and the results were analyzed by AlphaView SA.

### 2.8. Tube Formation and Migration of Endothelial Cells

96-well chambers were coated with 50 *μ*L of Matrigel (BD Biosciences) and placed at 37°C for 30 min. EA.hy926 cells (1.5 × 10^4^) were suspended in 150 *μ*L serum-free H-DMEM containing hucMSC-exosomes (100 *μ*g/mL) and plated under the Matrigel. Control wells contained 150 *μ*L serum-free H-DMEM with equal PBS. After being incubated in an atmosphere with 5% CO_2_ at 37°C for 10 h, three random fields were photographed under an inverted microscope and numbers of tubes were counted.

To test the migration of endothelial cells, EA.hy926 cells (2.5 × 10^4^) were suspended in 200 *μ*L serum-free H-DMEM and plated into the upper compartment of Transwell chambers (Corning, USA). Then the lower chamber was filled with 500 *μ*L serum-free H-DMEM containing hucMSC-exosomes (200 *μ*g/mL). Control wells contained 500 *μ*L serum-free H-DMEM with equal PBS. After culture in an atmosphere of humidified air with 5% CO_2_ at 37°C for 12 h, the cells remaining on top of the membrane were wiped off with a cotton swab, while the cells which had migrated through the membrane were fixed with 4% paraformaldehyde for 30 min and stained with crystal violet for 15 min. Finally, three random fields were photographed under an inverted microscope and cells were counted.

### 2.9. Statistical Analysis

Statistical analysis was performed using SPSS 16.0 software. Data are expressed as means ± standard deviation. Student's *t*-test was used to compare experimental and relative control groups. One-way ANOVA followed by a post hoc test was used to analyze variance among all groups. A value of *P* less than 0.05 was considered significant.

## 3. Results

### 3.1. Characterization of hucMSC-Exosomes

**Figure 2:**
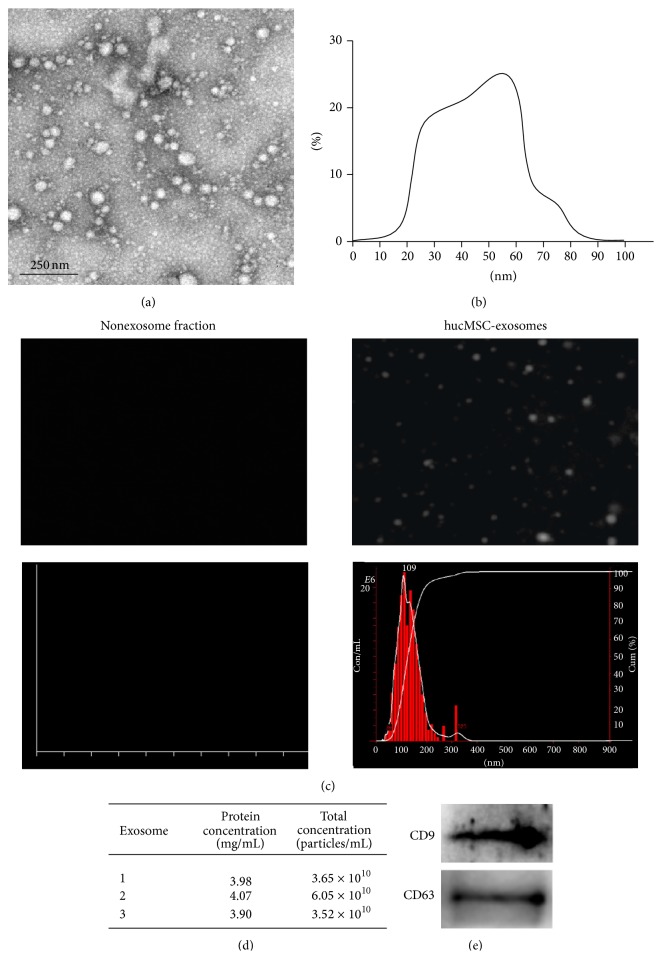
Identification of exosomes derived from hucMSC. Transmission electron photomicrograph of hucMSC-exosomes (a). Scale bar = 250 nm. Diameter ranges of hucMSC-exosomes under transmission electron microscopy (b). NTA of nonexosome fraction and hucMSC-exosomes (c). The protein concentration and particle concentration of hucMSC-exosomes in different batches (d). CD9 and CD63 expression in hucMSC-exosomes detected by western blot (e).

Transmission electron microscopic observation of hucMSC-exosomes revealed the presence of spherical vesicles, with a typical cup-shape. The size distribution profile displayed a homogeneous population from 20 to 85 nm (Figures 2(a) and 2(b)). The particle size distribution and particle pictorial diagram of hucMSC without exosomes (nonexosome) and exosomes were also recorded by NTA. There was no particle distribution in nonexosomes (Figure 2(c)). The mean protein concentration and mean particle concentration of hucMSC-exosomes were 3.98 mg/mL and 4.41 × 10^10^ particles/mL, respectively (Figure 2(d)). The isolated hucMSC-exosomes were found to express high levels of CD9 and CD63 (Figure 2(e)).

### 3.2. hucMSC-Exosomes Improved Cardiac Systolic Function in AMI Rats

**Figure 3:**
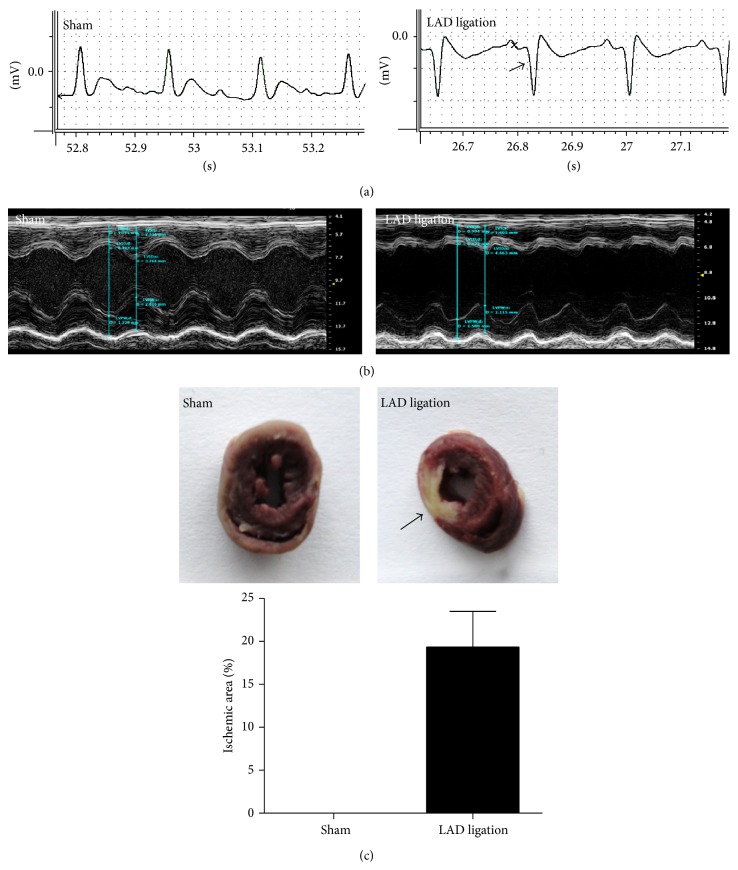
Establishment of a rat AMI model. Electrocardiogram of rats (a). A normal wave can be seen in the sham group. Arrow indicates the pathologic Q wave in an LAD ligated rat. The representative echocardiography (b). A sham rat shows a normal cardiograph. Contraction of the left ventricle anterior walls in the LAD ligated animal was weaker than in sham control animal. TTC staining of a myocardial section (c). The black arrow indicates the infarction area which occupied 19% in whole left ventricles.

AMI model was created by ligating LAD coronary artery. The results of a biological signal recording system showed that a visible pathologic Q wave was detected in the animals with LAD ligation as early as 24 h (Figure 3(a)). The cardiac function analysis by echocardiography showed that the contraction of the left ventricle anterior walls in the LAD ligated animals became weaker than that in sham control group (Figure 3(b)). TTC staining showed that part of the anterior wall of the left ventricle in the LAD ligated animal was pale, which represented the ischemic area that occupied 19% in whole left ventricles (Figure 3(c)).

**Figure 4:**
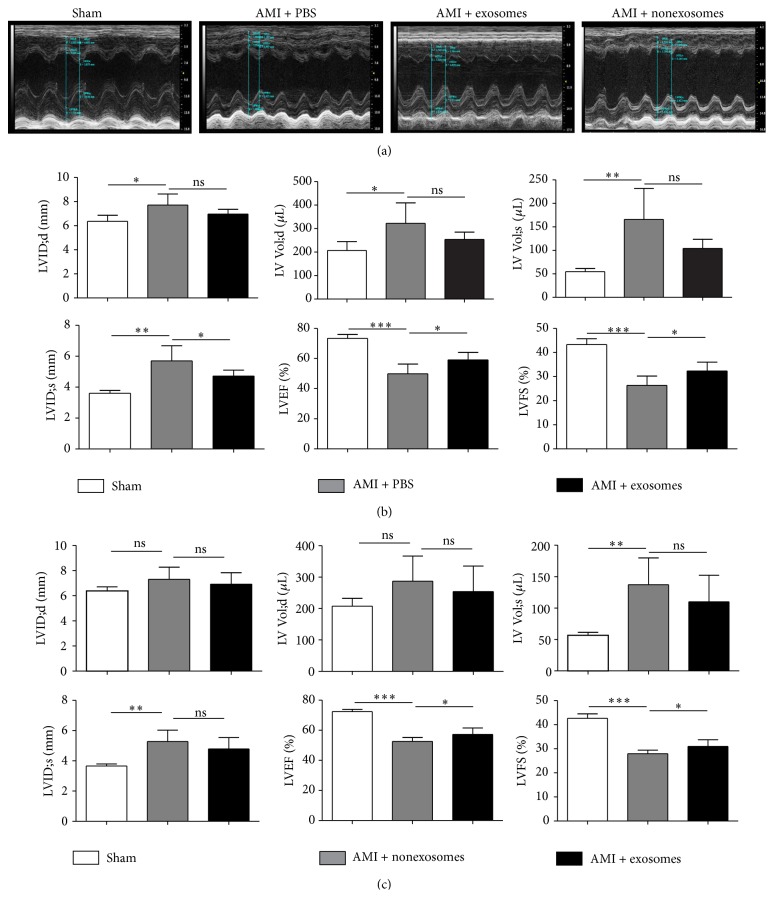
hucMSC-exosomes improved the cardiac systolic function of AMI rats 4 weeks after exosomes infusion. Echocardiographs (a). Cardiac function related parameters (LVEF, LVFS, LVID;s, LVID;d; LV Vol;d, and LV Vol;s) in hucMSC-exosomes (b) and in hucMSC-CM without exosomes treated animals (c). ^*∗*^ *P* < 0.05, ^*∗∗*^ *P* < 0.01, and ^*∗∗∗*^ *P* < 0.001; ns: no significant difference between the two groups.

To determine the effect of hucMSC-exosomes, cardiac function was analyzed using echocardiography four weeks after infusion of hucMSC-exosomes. The echocardiogram in the parasternal long axis view showed that the contraction of the left ventricle anterior walls was stronger in the exosome-treated animals compared to that treated with PBS or nonexosome fraction (Figure 4(a)). We further evaluated LVEF, LVFS, LVID;d, LVID;s, LV Vol;d, and LV Vol;s. Being compared to the animals in sham group, LVEF and LVFS were both significantly decreased in AMI + PBS treated animals (*P* < 0.001), while LVID;s, LVID;d, LV Vol;d, and LV Vol;s were all significantly increased in the AMI+PBS group [LVID;d and LV Vol;d (*P* < 0.05), LVID;s and LV Vol;s (*P* < 0.01)]. However, LVEF and LVFS were both increased significantly in the exosome-treated group compared to the PBS control group (*P* < 0.05), while LVID;s in the exosome-treated group was lower than in the PBS control group (*P* < 0.05). There was no significant difference between exosome-treated and PBS-treated animals in LV Vol;s, LVID;d, or LV Vol;d (Figure 4(b)). In order to further confirm the significance and specificity of exosomes, the related parameters were evaluated in the animals infused with hucMSC-CM without exosomes four weeks later. The results showed that LVEF and LVFS were both significantly decreased in the animals treated with AMI + nonexosomes compared to the animals treated with exosomes (*P* < 0.05) (Figure 4(c)).

### 3.3. hucMSC-Exosomes Reduced Cardiac Fibrosis, Suppressed Cell Apoptosis, and Promoted Cell Proliferation

**Figure 5:**
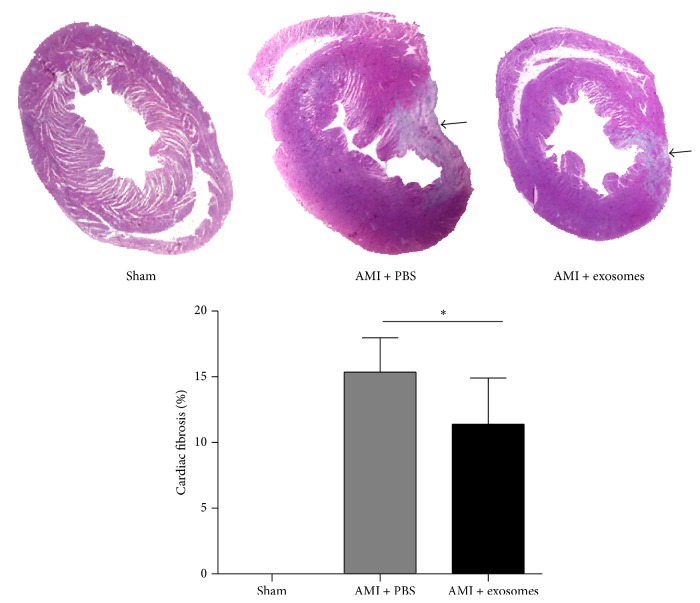
hucMSC-exosomes reduced cardiac fibrosis. Representative micrographs of sections of the left ventricles stained with Masson's trichrome and the percentage of cardiac fibrosis. Arrows indicate the fibrotic myocardial tissue. ^*∗*^ *P* < 0.05 versus AMI + PBS.

**Figure 6:**
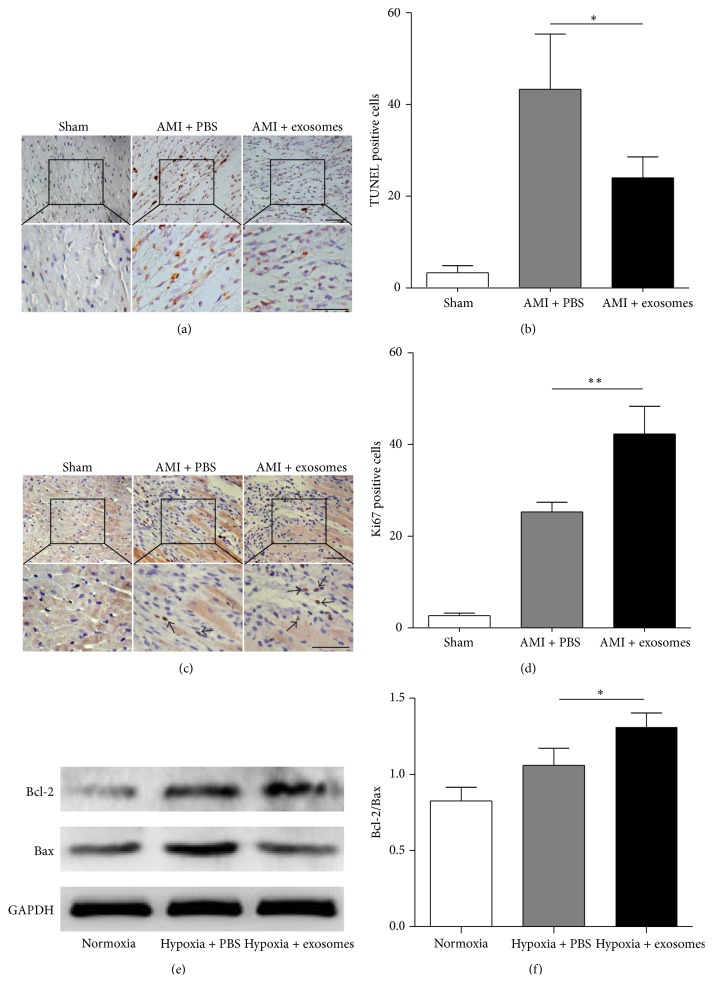
hucMSC-exosomes reduced cell apoptosis and promoted cell proliferation. The apoptotic cells in the ischemic zone of myocardial tissue sections (a). Scale bar = 50 *μ*m. The numbers of TUNEL positive cells in 3 randomly selected fields at 200 times magnification for each rat (b). ^*∗*^ *P* < 0.05 versus AMI + PBS. Immunohistochemical staining of Ki67 expression in myocardial tissue from the border zone of an infarcted left ventricle (c). The black arrows indicate Ki67 positive cells. Scale bar = 50 *μ*m. The numbers of Ki67 positive cells in 3 randomly selected fields at 200 times magnification for each rat (d). ^*∗∗*^ *P* < 0.01 versus AMI + PBS. The protein of Bax and Bcl-2 in the myocardial cells H9C2(2-1) cultured under hypoxic conditions was evaluated by western blot (e). Statistical analysis of the ratio of Bcl-2 to Bax (f). ^*∗*^ *P* < 0.05 versus Hypoxia + PBS.

Masson's trichrome staining revealed that no blue areas were visible in the sham group, while the percentage of cardiac fibrosis in the exosome-treated group was significantly less than that in the PBS control group (Figure 5). Cardiac repair is closely associated with a reduction in cardiomyocyte apoptosis. There were fewer apoptotic cells, stained with TUNEL, in the myocardium of the exosome-treated group compared with the PBS-treated group (Figures 6(a) and 6(b)). Moreover, Ki67 positive cells were detected from the border zone of infarcted left ventricles by immunohistochemical staining. Ki67 positive cells in exosome-treated animals were significantly increased compared to that in PBS-treated group (Figures 6(c) and 6(d)).

To investigate the antiapoptotic effect of hucMSC-exosomes, the expression of Bax and Bcl-2 proteins in the rat myocardial cells H9C2(2-1) was detected using western blot. Following exposure to hypoxic conditions for 12 h, the ratio of Bcl-2 to Bax in exosome-treated H9C2(2-1) cells was significantly higher than that in control cells (Figures 6(e) and 6(f)).

### 3.4. hucMSC-Exosomes Promoted Angiogenesis

**Figure 7:**
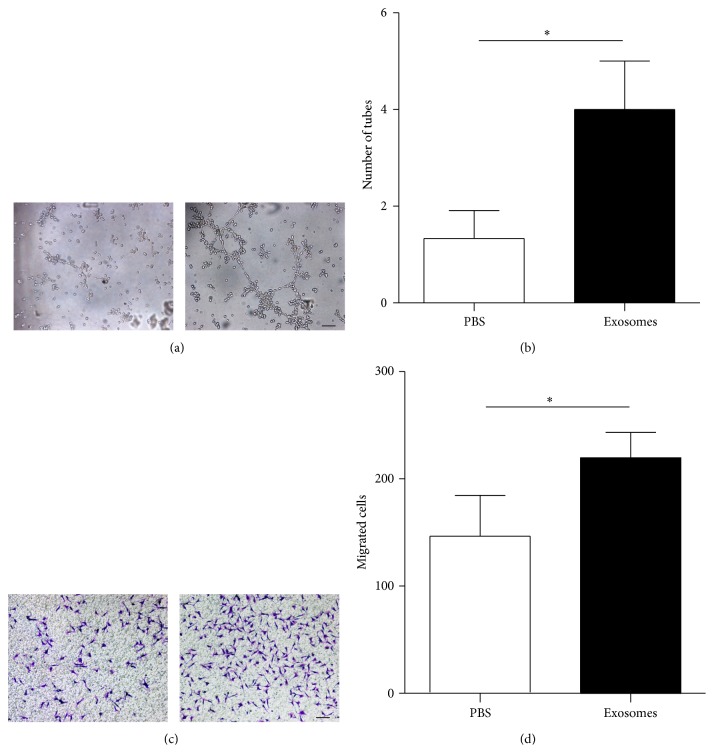
hucMSC-exosomes accelerated tube-like structure formation and migration of EA.hy926 cells. Representative photomicrographs of tube-like structure (a) and quantification of tube number (b). Scale bar = 100 *μ*m. ^*∗*^ *P* < 0.05 versus PBS control. Representative photomicrographs of EA.hy926 cells which migrated through the filter, stained with crystal violet (c). Quantification of cells migrated through the Transwell membrane (d). Scale bar = 100 *μ*m. ^*∗*^ *P* < 0.05 versus PBS control.

Myocardial repair and the improvement of cardiac systolic function may be related to a promotion of angiogenesis.* In vitro* study showed that tube-like structure was significantly more in exosomes treated EA.hy926 cells than that in control (*P* < 0.05) (Figures 7(a) and 7(b)). Moreover, the migration of EA.hy926 cells in the hucMSC-exosome-treated group was significantly greater than that in the PBS-treated group (*P* < 0.05) after incubation for 12 h (Figures 7(c) and 7(d)).

## 4. Discussion

In this study, we successfully isolated and characterized exosomes from hucMSC. We demonstrated that administration of hucMSC-exosomes (i.v.) following AMI significantly increased LVEF and LVFS and reduced cardiac fibrosis. However, hucMSC-CM without exosomes shows hardly any effect on prompting cardiac function recovery, suggesting hucMSC-exosomes mediated myocardium regeneration. Moreover, hucMSC-exosomes prevented cardiomyocyte apoptosis and promoted cell proliferation in the border zone. Furthermore, the exosomes promoted myocardial repair that also is related to promotion of angiogenesis.* In vitro* we demonstrated that hucMSC-exosomes could promote tube formation and migration of EA.hy926 cells.

It is well known that the exosomal contents comprise mRNA, DNA, microRNAs, proteins (i.e, enzymes, growth factors, and cytokines), and transmembrane proteins of different kinds, including tetraspanins, annexin, and intracellular cell adhesion molecule. The proteins or miRNAs carried by hucMSC-exosomes may be associated with the promotion of cell protection and angiogenesis. It has been reported that several types of cells, for example, cardiomyocyte [35] and endothelial cells [36], can take up exosomes. In our study, exosomes were injected through tail vein. They would be very quickly moved forward following blood flow. Therefore, the possibility to be uptaken by tail endothelial cells or fused with these cells was very low. However, when exosomes reached a tissue or organ at which blood flow was very slow, for example, the board area of ischemic myocardium, the movement of exosomes might be slowed down, which might significantly increase the chance to be internalized by the survived cells or fused with these cells. Therefore, exosomes can transfer molecules from one cell to another via membrane vesicle trafficking and may play in cell-to-cell signaling, so it is often hypothesized that delivery of their cargo RNA molecules can explain biological effects. It has been indicated that the exosomes can transfer proteases in the treatment of myocardial ischemia-reperfusion injury [37].

Our results in this study suggest that hucMSC-exosomes significantly increased Bcl-2 in cardiomyocytes following exposure to low oxygen environment. Bcl-2 is specifically considered as an important antiapoptotic protein. The results from Arslan et al. also showed that MSC-derived exosomes increased ATP levels, decreased oxidative stress, and activated the PI3K/Akt pathway to enhance myocardial viability and prevent adverse remodeling after myocardial ischemia/reperfusion injury [38].

In addition, hucMSC-exosomes also increased Ki67 expression in the border zone of AMI rats. The Ki67 protein is strictly associated with cell proliferation. To identify the type of the proliferated cells, we are planning to do double stain with cardiomyocyte markers, for example, *α*-actinin and Ki67, to confirm that the proliferated cells are cardiomyocyte.

In conclusion, hucMSC-exosomes promoted heart repair following ischemic injury through protecting myocardial cells from apoptosis and promoting cell proliferation and angiogenesis. This study may provide a new mechanism by which hucMSC can be effectively used for clinical treatment of AMI.

## References

[1] Hynes B., Kumar A. H. S., O'Sullivan J. (2013). Potent endothelial progenitor cell-conditioned media-related anti-apoptotic, cardiotrophic, and pro-angiogenic effects post-myocardial infarction are mediated by insulin-like growth factor-1. *European Heart Journal*.

[2] Valina C., Pinkernell K., Song Y.-H. (2007). Intracoronary administration of autologous adipose tissue-derived stem cells improves left ventricular function, perfusion, and remodelling after acute myocardial infarction. *European Heart Journal*.

[3] van den Akker F., Deddens J. C., Doevendans P. A., Sluijter J. P. G. (2013). Cardiac stem cell therapy to modulate inflammation upon myocardial infarction. *Biochimica et Biophysica Acta*.

[4] Rota M., Padin-Iruegas M. E., Misao Y. (2008). Local activation or implantation of cardiac progenitor cells rescues scarred infarcted myocardium improving cardiac function. *Circulation Research*.

[5] Schuleri K. H., Feigenbaum G. S., Centola M. (2009). Autologous mesenchymal stem cells produce reverse remodelling in chronic ischaemic cardiomyopathy. *European Heart Journal*.

[6] Hare J. M., Traverse J. H., Henry T. D. (2009). A randomized, double-blind, placebo-controlled, dose-escalation study of intravenous adult human mesenchymal stem cells (prochymal) after acute myocardial infarction. *Journal of the American College of Cardiology*.

[7] Vassalli G., Moccetti T. (2011). Cardiac repair with allogeneic mesenchymal stem cells after myocardial infarction. *Swiss Medical Weekly*.

[8] Shake J. G., Gruber P. J., Baumgartner W. A. (2002). Mesenchymal stem cell implantation in a swine myocardial infarct model: engraftment and functional effects. *The Annals of Thoracic Surgery*.

[9] Makkar R. R., Price M. J., Lill M. (2005). Intramyocardial injection of allogenic bone marrow-derived mesenchymal stem cells without immunosuppression preserves cardiac function in a porcine model of myocardial infarction. *Journal of Cardiovascular Pharmacology and Therapeutics*.

[10] Kawada H., Fujita J., Kinjo K. (2004). Nonhematopoietic mesenchymal stem cells can be mobilized and differentiate into cardiomyocytes after myocardial infarction. *Blood*.

[11] Nagaya N., Kangawa K., Itoh T. (2005). Transplantation of mesenchymal stem cells improves cardiac function in a rat model of dilated cardiomyopathy. *Circulation*.

[12] Hass R., Kasper C., Böhm S., Jacobs R. (2011). Different populations and sources of human mesenchymal stem cells (MSC): a comparison of adult and neonatal tissue-derived MSC. *Cell Communication and Signaling*.

[13] Pelekanos R. A., Li J., Gongora M. (2012). Comprehensive transcriptome and immunophenotype analysis of renal and cardiac MSC-like populations supports strong congruence with bone marrow MSC despite maintenance of distinct identities. *Stem Cell Research*.

[14] Chong J. J. H., Chandrakanthan V., Xaymardan M. (2011). Adult cardiac-resident MSC-like stem cells with a proepicardial origin. *Cell Stem Cell*.

[15] Baksh D., Yao R., Tuan R. S. (2007). Comparison of proliferative and multilineage differentiation potential of human mesenchymal stem cells derived from umbilical cord and bone marrow. *Stem Cells*.

[16] Weiss M. L., Anderson C., Medicetty S. (2008). Immune properties of human umbilical cord Wharton's jelly-derived cells. *Stem Cells*.

[17] Chen Y., Qian H., Zhu W. (2011). Hepatocyte growth factor modification promotes the amelioration effects of human umbilical cord mesenchymal stem cells on rat acute kidney injury. *Stem Cells and Development*.

[18] Cao H., Qian H., Xu W. (2010). Mesenchymal stem cells derived from human umbilical cord ameliorate ischemia/reperfusion-induced acute renal failure in rats. *Biotechnology Letters*.

[19] Yan Y., Xu W., Qian H. (2009). Mesenchymal stem cells from human umbilical cords ameliorate mouse hepatic injury in vivo. *Liver International*.

[20] Qian H., Yang H., Xu W. (2008). Bone marrow mesenchymal stem cells ameliorate rat acute renal failure by differentiation into renal tubular epithelial-like cells. *International Journal of Molecular Medicine*.

[21] Rota M., Kajstura J., Hosoda T. (2007). Bone marrow cells adopt the cardiomyogenic fate in vivo. *Proceedings of the National Academy of Sciences of the United States of America*.

[22] Xu M., Wani M., Dai Y.-S. (2004). Differentiation of bone marrow stromal cells into the cardiac phenotype requires intercellular communication with myocytes. *Circulation*.

[23] Miyahara Y., Nagaya N., Kataoka M. (2006). Monolayered mesenchymal stem cells repair scarred myocardium after myocardial infarction. *Nature Medicine*.

[24] Wang L., Deng J., Tian W. (2009). Adipose-derived stem cells are an effective cell candidate for treatment of heart failure: an MR imaging study of rat hearts. *The American Journal of Physiology—Heart and Circulatory Physiology*.

[25] Gnecchi M., Zhang Z., Ni A., Dzau V. J. (2008). Paracrine mechanisms in adult stem cell signaling and therapy. *Circulation Research*.

[26] Kuraitis D., Ruel M., Suuronen E. J. (2011). Mesenchymal stem cells for cardiovascular regeneration. *Cardiovascular Drugs and Therapy*.

[27] Timmers L., Lim S. K., Arslan F. (2008). Reduction of myocardial infarct size by human mesenchymal stem cell conditioned medium. *Stem Cell Research*.

[28] Lai R. C., Arslan F., Lee M. M. (2010). Exosome secreted by MSC reduces myocardial ischemia/reperfusion injury. *Stem Cell Research*.

[29] Lai R. C., Chen T. S., Lim S. K. (2011). Mesenchymal stem cell exosome: a novel stem cell-based therapy for cardiovascular disease. *Regenerative Medicine*.

[30] Li T., Yan Y., Wang B. (2013). Exosomes derived from human umbilical cord mesenchymal stem cells alleviate liver fibrosis. *Stem Cells and Development*.

[31] Zhou Y., Xu H., Xu W. (2013). Exosomes released by human umbilical cord mesenchymal stem cells protect against cisplatin-induced renal oxidative stress and apoptosis in vivo and in vitro. *Stem Cell Research & Therapy*.

[32] Zhang B., Wang M., Gong A. (2015). HucMSC-exosome mediated-wnt4 signaling is required for cutaneous wound healing. *Stem Cells*.

[33] Qiao C., Xu W., Zhu W. (2008). Human mesenchymal stem cells isolated from the umbilical cord. *Cell Biology International*.

[34] Qu J.-L., Qu X.-J., Zhao M.-F. (2009). Gastric cancer exosomes promote tumour cell proliferation through PI3K/Akt and MAPK/ERK activation. *Digestive and Liver Disease*.

[35] Yu B., Kim H. W., Gong M. (2015). Exosomes secreted from GATA-4 overexpressing mesenchymal stem cells serve as a reservoir of anti-apoptotic microRNAs for cardioprotection. *International Journal of Cardiology*.

[36] Bian S., Zhang L., Duan L., Wang X., Min Y., Yu H. (2014). Extracellular vesicles derived from human bone marrow mesenchymal stem cells promote angiogenesis in a rat myocardial infarction model. *Journal of Molecular Medicine*.

[37] Lai R. C., Tan S. S., Teh B. J. (2012). Proteolytic potential of the msc exosome proteome: implications for an exosome-mediated delivery of therapeutic proteasome. *International Journal of Proteomics*.

[38] Arslan F., Lai R. C., Smeets M. B. (2013). Mesenchymal stem cell-derived exosomes increase ATP levels, decrease oxidative stress and activate PI3K/Akt pathway to enhance myocardial viability and prevent adverse remodeling after myocardial ischemia/reperfusion injury. *Stem Cell Research*.

